# Transcriptomes of human primary skin fibroblasts of healthy individuals reveal age‐associated mRNAs and long noncoding RNAs

**DOI:** 10.1111/acel.13915

**Published:** 2023-07-18

**Authors:** Dimitrios Tsitsipatis, Jennifer L. Martindale, Krystyna Mazan‐Mamczarz, Allison B. Herman, Yulan Piao, Nirad Banskota, Jen‐Hao Yang, Linna Cui, Carlos Anerillas, Ming‐Wen Chang, Mary Kaileh, Rachel Munk, Xiaoling Yang, Ceereena Ubaida‐Mohien, Chee W. Chia, Ajoy C. Karikkineth, Linda Zukley, Jarod D'Agostino, Kotb Abdelmohsen, Nathan Basisty, Supriyo De, Luigi Ferrucci, Myriam Gorospe

**Affiliations:** ^1^ Laboratory of Genetics and Genomics, National Institute on Aging Intramural Research Program National Institutes of Health Baltimore Maryland USA; ^2^ Translational Gerontology Branch, National Institute on Aging Intramural Research Program National Institutes of Health Baltimore Maryland USA; ^3^ Laboratory of Molecular Biology and Immunology, National Institute on Aging Intramural Research Program National Institutes of Health Baltimore Maryland USA; ^4^ Clinical Research Core, National Institute on Aging Intramural Research Program National Institutes of Health Baltimore Maryland USA

**Keywords:** aging, circular RNAs, human dermal fibroblasts, long noncoding RNAs, messenger RNAs, transcriptome

## Abstract

Changes in the transcriptomes of human tissues with advancing age are poorly cataloged. Here, we sought to identify the coding and long noncoding RNAs present in cultured primary skin fibroblasts collected from 82 healthy individuals across a wide age spectrum (22–89 years old) who participated in the GESTALT (Genetic and Epigenetic Signatures of Translational Aging Laboratory Testing) study of the National Institute on Aging, NIH. Using high‐throughput RNA sequencing and a linear regression model, we identified 1437 coding RNAs (mRNAs) and 1177 linear and circular long noncoding (lncRNAs) that were differentially abundant as a function of age. Gene set enrichment analysis (GSEA) revealed select transcription factors implicated in coordinating the transcription of subsets of differentially abundant mRNAs, while long noncoding RNA enrichment analysis (LncSEA) identified RNA‐binding proteins predicted to participate in the age‐associated lncRNA profiles. In summary, we report age‐associated changes in the global transcriptome, coding and noncoding, from healthy human skin fibroblasts and propose that these transcripts may serve as biomarkers and therapeutic targets in aging skin.

## Graphical abstract

Changes in the transcriptomes of different cell types with advancing human age are poorly characterized. Here, we used RNA‐sequencing analysis to identify the RNAs expressed by primary skin fibroblasts collected from 82 healthy individuals across a wide age spectrum. We identified and validated many coding RNAs (mRNAs) and long noncoding RNAs (lncRNAs) changing with skin fibroblast aging, which could represent valuable biomarkers and therapeutic targets.

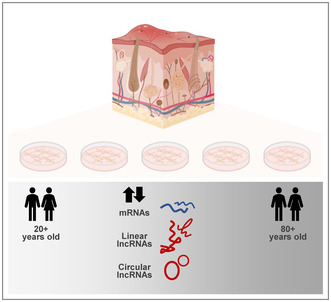

**Abbreviations**
ANXA2R: annexin a2 receptor
AXIN2: Axin 2
B2M: beta‐2‐microglobulin
COL5A3: collagen type V alpha 3 chain
CTGF/CCN2: connective tissue growth factor/cellular communication network factor 2
DHODH: dihydroorotate dehydrogenase
ECM: extracellular matrix
FASTKD2: FAS‐activated serine/threonine kinase D2
FGF9: growth factor 9
FOX: forkhead box
GATA3: GATA‐binding protein 3
GESTALT: Genetic and Epigenetic Signatures of Translational Aging Laboratory Testing
GSEA: gene set enrichment analysis
HDF: human dermal fibroblast
HMGB1: high mobility group protein‐1
HNF: hepatocyte nuclear factor
HNRNPC: heterogeneous nuclear ribonucleoprotein C
HOX: homeobox
HSF1: heat shock factor 1
ILF3: interleukin enhancer‐binding factor 3
IRF1: interferon regulatory factor 1
LEF1: lymphoid enhancer‐binding factor 1
lncRNAs: long noncoding RNAs
LncSEA: LncRNA set enrichment analysis
LYVE1: lymphatic vessel endothelial hyaluronan receptor 1
MAIP1: matrix AAA peptidase interacting protein 1
mRNAs: messenger RNAs
OLFM1: olfactomedin‐1
PAX4: paired box 4
PBX1: pre‐B‐cell leukemia homeobox 1
PCBP2: poly(C)‐binding protein
PCSK2: proprotein convertase subtilisin/kexin type 2
PIT1: pituitary‐specific positive transcription factor 1
PLS: partial least square
RBM: RNA‐binding motif protein
RBPs: RNA‐binding proteins
RNA‐seq: RNA sequencing
RNFT2: RING finger transmembrane domain‐containing protein 2
RT‐qPCR: reverse transcription followed by real‐time quantitative PCR
SMAD: mothers against decapentaplegic homolog
SNAI2: snail family transcriptional repressor 2
TF: transcription factor
TGF‐β, TGFB1: transforming growth factor‐beta
TGIF: TGFB‐induced factor homeobox
ZNF362: zinc finger protein 362

## 1 INTRODUCTION

With advancing age, changes in gene expression programs in cells across the body are reflected in the aging phenotypes of tissues and organs. Therefore, interest in elucidating the RNAs and proteins that govern cell function as aging progresses has recently intensified, with the hope of shedding light on the processes that underlie both healthy aging and age‐related disease. Aging of the outermost barrier in our body, the skin, can compromise skin integrity and increase the risk of damage and infection of internal organs (Kim et al., 2023; Zhang & Duan, 2018). With aging, the fibroblasts in the dermis—the skin layer derived from the mesoderm—experience reductions in number and function, including the capacity to synthesize and remodel the extracellular matrix (Braverman, 2000; Makrantonaki & Zouboulis, 2007; Shuster et al., 1975).

We recently reported the proteomes of skin fibroblasts derived from chronologically aged healthy individuals (Tsitsipatis et al., 2022). To gain a complementary view of this process, we expanded our studies to the transcriptome, focusing on both coding messenger RNAs (mRNAs) and long noncoding RNAs (lncRNAs). Skin punch biopsies were obtained from the inner axilla, an area that is typically not exposed to sunlight (Fisher et al., 2002; McCabe et al., 2020), from 82 individual participants spanning a wide age range [22–89 years old (y.o.)] who were evaluated as healthy according to the stringent clinical and functional criteria of the Genetic and Epigenetic Signatures of Translational Aging Laboratory Testing (GESTALT) study of the National Institute on Aging (NIA), NIH (Tanaka et al., 2018; Tsitsipatis et al., 2022; Tumasian et al., 2021). High‐throughput RNA sequencing (RNA‐seq) analysis using linear and spline regression models uncovered numerous mRNAs as well as linear and circular lncRNAs differentially expressed as a function of participant age. Gene set enrichment analysis (GSEA) and LncRNA set enrichment analysis (LncSEA) (J. Chen et al., 2021; Subramanian et al., 2005) identified several transcription factors (TFs), including members of both the forkhead box (FOX) and homeobox (HOX) TF families, as well as splicing RNA‐binding proteins (RBPs). We propose that these TFs and RBPs may foster changes in the transcriptome with age. Given the limited knowledge of lncRNA functions in human physiology, this study identifies promising molecular factors implicated in the aging of skin cells.

## 2 RESULTS

### 2.1 Transcriptomic analysis of primary skin fibroblasts from biopsies of GESTALT donors aged 22–89 years old

**FIGURE 1:**
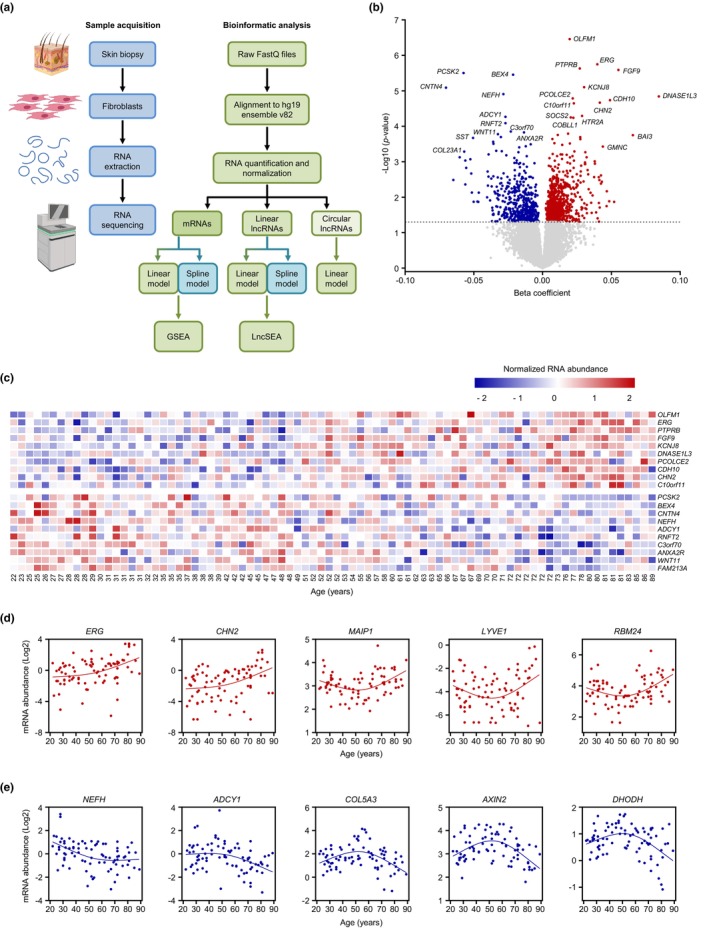
Differentially expressed coding transcripts (mRNAs) with advanced aging. (a) Workflow of sample acquisition and preparation (blue) and bioinformatic analysis (green) followed in this study. (b) Volcano plot showing beta coefficients of mRNAs expressed with age (per year). Transcripts showing significantly increased (red dots) or decreased (blue dots) levels with age (*p* < 0.05, adjusted for covariates) are indicated. Gray dots show transcripts that did not change significantly with age (*p* > 0.05). (c) Heatmap of the top 10 significantly elevated mRNAs (*top*) and top 10 significantly reduced mRNAs (*bottom*) with age based on a linear regression model (unadjusted *p*‐values). (d,e) Graphs of mRNAs showing differentially increased (d) or decreased (e) slopes after 50 years of age based on a spline regression model (unadjusted *p*‐values). In (b), the *R* ^2^ values for the reported transcripts increasing with advancing age are as follows: *R* ^2^ = 0.25 for *OLFM1* mRNA; *R* ^2^ = 0.14 for *ERG* mRNA; *R* ^2^ = 0.21 for *PTPRB* mRNA; *R* ^2^ = 0.14 for *FGF9* mRNA; *R* ^2^ = 0.16 for *KCNJ8* mRNA; *R* ^2^ = 0.081 for *DNASE1L3* mRNA; *R* ^2^ = 0.16 for *PCOLE2* mRNA; *R* ^2^ = 0.16 for *CDH10* mRNA; *R* ^2^ = 0.13 for *CHN2* mRNA; and *R* ^2^ = 0.14 for *C10orf11* mRNA. Similarly, for the reported transcripts decreasing with advancing age, the *R* ^2^ values are as follows: *R* ^2^ = 0.13 for *PCSK2* mRNA; *R* ^2^ = 0.18 for *BEX4* mRNA; *R* ^2^ = 0.11 for *CNTN4* mRNA; *R* ^2^ = 0.15 for *NEFH* mRNA; *R* ^2^ = 0.11 for *ADCY1* mRNA; *R* ^2^ = 0.15 for *RNFT2* mRNA; *R* ^2^ = 0.18 for *C3orf70* mRNA; *R* ^2^ = 0.18 for *ANAX2R* mRNA; *R* ^2^ = 0.081 for *WNT11* mRNA; and *R* ^2^ = 0.13 for *FAM213A* mRNA.

Primary skin fibroblasts were established and expanded from biopsies collected from 82 healthy individuals in the GESTALT cohort (Table 1; NIA, NIH). Total RNA was then extracted from these skin fibroblasts and ribosomal (r)RNA‐depleted samples were subjected to high‐throughput paired‐end RNA sequencing (RNA‐seq) (Figure 1a, *Sample acquisition*) (Methods); the RNA‐seq data were deposited in GSE226189. Following alignment to the human genome, normalization, and quantification, the transcriptomic profiles were divided into coding [messenger RNAs (mRNAs)] and linear or circular long noncoding RNAs (lncRNAs); we then employed linear and spline models to identify differentially expressed transcripts, and we used the GSEA and LncSEA methods (J. Chen et al., 2021; Subramanian et al., 2005) to find molecules interacting with the differentially expressed RNAs (Figure 1a, *Bioinformatic Analysis*).

**TABLE 1:** Demographic data of the individuals who participated in the study.

	Female	Male
Number	35	47
Average age (years)^a^	54 ± 17	52 ± 20
Age range (years)	25–80	22–89
Race	32 Caucasian	36 Caucasian
	1 African American	10 African American
	2 Asian	1 Asian

*Note*: Demographic data for the very healthy individuals in the GESTALT study [inclusion criteria described in (Tsitsipatis et al., 2022)] who provided skin biopsies for this study. After expansion, the skin fibroblasts derived from these healthy individuals were used for RNA‐seq analysis and validation.

^a^ Average age (± SD) at time of skin biopsy collection.

Using a linear regression model, we found that out of the 16,455 mRNAs expressed in this cohort, 1437 showed significant change with age (unadjusted *p*‐value <0.05) (Figure 1b and Appendix S1, *mRNAs—linear model*). Most of these mRNAs increased with age, as seen for olfactomedin‐1 (*OLFM1*) mRNA, which encodes a protein with enhanced secretion in aged primary skin fibroblasts (Waldera Lupa et al., 2015), and fibroblast growth factor 9 (*FGF9*) mRNA, encoding a protein that inhibits myogenesis as well as myofibroblast differentiation in idiopathic pulmonary fibrosis (Huang et al., 2019; Joannes et al., 2016). Among the handful of reduced mRNAs, RING finger transmembrane domain‐containing protein 2 (*RNFT2*) mRNA, encoding a protein that represses interleukin 3 (IL3) signaling (Tong et al., 2020), and *WNT11* mRNA, encoding a protein that is less abundant in old mouse liver (Hofmann et al., 2014), were previously associated with aging (Figure 1c). Notably, a subset of the top mRNAs that were differentially abundant with age, including those that encode OLFM1, FGF9, annexin a2 receptor (ANXA2R), and proprotein convertase subtilisin/kexin type 2 (PCSK2), followed the same trajectory in human lung fibroblasts WI‐38 and IMR‐90 rendered senescent by replicative exhaustion (Casella et al., 2019).

By employing the spline interpolation model, we identified 1436 mRNAs differentially expressed with age using an unadjusted *p*‐value <0.05 (Appendix S1, *mRNAs—spline model*). While most of these were also identified by the linear regression model, a handful of these mRNAs that did not change linearly with age were detected only when using the spline model. They included mRNAs for which the abundance slope increased after 50 years of age [e.g., those encoding matrix AAA peptidase interacting protein 1 (MAIP1, also known as C2ORF47), lymphatic vessel endothelial hyaluronan receptor 1 (LYVE1), and RNA‐binding motif protein 24 (RBM24) (Figure 1d)] or decreased after 50 years of age [e.g., collagen type V alpha 3 chain (*COL5A3*), axin 2 (*AXIN2*), and dihydroorotate dehydrogenase (*DHODH*) mRNAs (Figure 1e)].

Given that hormones such as estradiol and testosterone can modulate the production of major components in skin, such as hydroxyproline (Brincat et al., 1983), we sought to identify how the transcriptome may change separately in females and males as a function of healthy aging. Using the linear regression model, we identified 1425 and 476 differentially abundant coding transcripts (mRNAs) using an unadjusted *p*‐value <0.05 with age in females (Figure S1a and Appendix S1, *coding—linear—females*) and males (Figure S1b and Appendix S1, *coding—linear—males*), respectively. Interestingly, using the spline model (unadjusted *p*‐value <0.05), the number of differentially abundant mRNAs did not change significantly in males (313 mRNAs; Figure S1d and Appendix S1, *coding—spline—males*), but it increased significantly in females (4133 mRNAs; Figure S1c and Appendix S1, *coding—spline—females*). Notably, *NOG* mRNA, encoding the secreted protein noggin (NOG), which antagonizes members of the transforming growth factor‐β (TGF‐β) family (Groppe et al., 2002; Massague, 2012), was the top differentially abundant mRNA in females when using either the linear or the spline regression model.

### 2.2 Subsets of differentially abundant mRNAs in skin fibroblasts from older donors are putative transcriptional targets of FOX and HOX transcription factors

**FIGURE 2:**
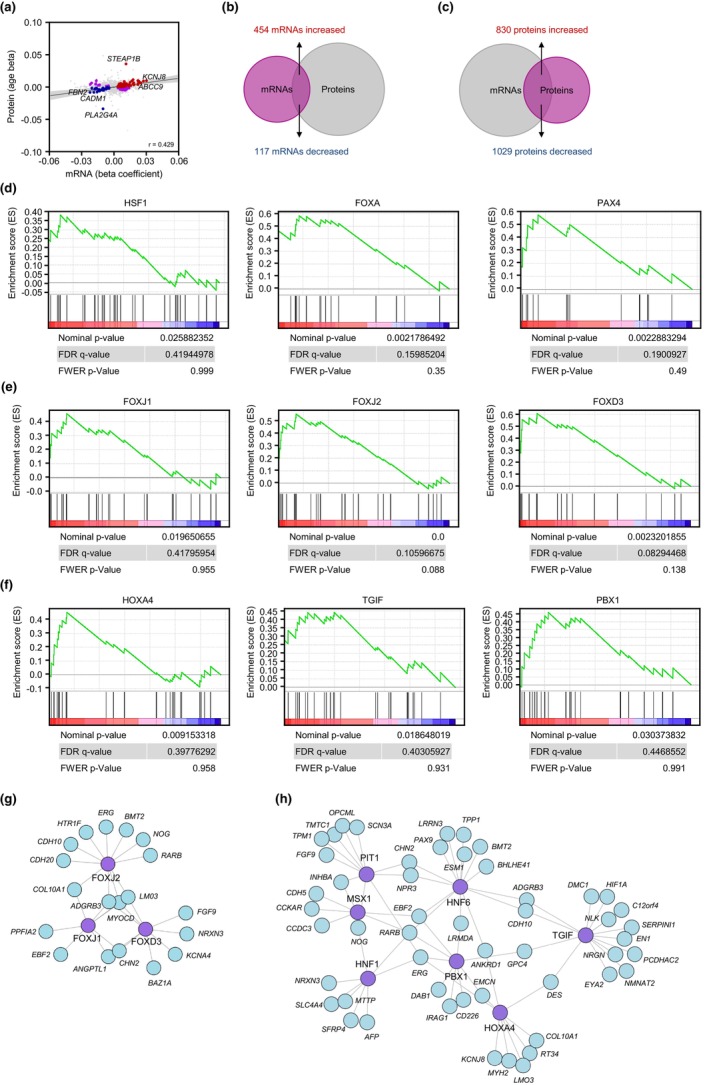
Transcription factors (TFs) putatively driving the expression of differentially abundant mRNAs with age based on a linear regression model. (a) Correlations between differentially abundant mRNAs (unadjusted *p*‐value <0.05) and differentially abundant proteins (unadjusted *p*‐value <0.05; Tsitsipatis et al., 2022) from the same cohort as a function of age. Proteins and mRNAs changing in the same direction are indicated with red dots (both increased) or blue dots (both decreased), while proteins and mRNAs changing in opposite directions are indicated in purple dots (Pearson correlation coefficient, *r* = 0.429). Gray dots represent instances in which either the mRNA levels or the protein levels did not change significantly (unadjusted *p*‐value >0.05). (b) Overlap of differentially abundant mRNAs (unadjusted *p*‐value <0.05) for which the levels of encoded proteins were not significantly changed (unadjusted *p*‐value >0.05). (c) Overlap of differentially abundant proteins (unadjusted *p*‐value <0.05) encoded by mRNAs that did not show significant changes in abundance (unadjusted *p*‐value >0.05). (d) TFs HSF1, FOXA, and PAX4 (above the graphs), previously associated with longevity, capable of transcribing mRNAs that were enriched with age by the GSEA method. (e,f) TFs in the FOX (e) or HOX (f) families whose transcribed mRNAs were preferentially elevated in older individuals by the GSEA method. (g,h) Network of differentially expressed mRNAs, predicted to be transcriptionally induced by the FOX (e) or HOX (f) transcription factors.

To begin to investigate if the changes in the transcriptome with aging were reflected in changes in the proteome (adjusted for all covariates), we compared the differentially abundant mRNAs identified using the linear regression model (unadjusted *p*‐value <0.05) with the differentially abundant proteins (unadjusted *p*‐value <0.05) we previously reported in the same skin fibroblasts from this cohort (Tsitsipatis et al., 2022). At the overlap of these two datasets (Appendix S2), we found that the differentially expressed mRNAs (beta coefficient) modestly correlated with the differentially expressed proteins (age beta) as a function of age (Figure 2a; Pearson correlation coefficient, *r* = 0.429). We expanded this analysis by identifying differentially expressed mRNAs (unadjusted *p*‐value <0.05) for which the levels of the encoded proteins were not significantly changed (unadjusted *p*‐value >0.05); 454 such mRNAs were increased, whereas 117 were decreased (Figure 2b). Conversely, we identified differentially expressed proteins (unadjusted *p*‐value <0.05) for which the levels of the encoding mRNAs were not significantly changed (unadjusted *p*‐value >0.05); we found 830 and 1029 such proteins that either increased or decreased, respectively (Figure 2c).

We then asked whether the transcriptomic changes may be jointly regulated by shared transcription factors (TFs) in this paradigm. We used the GSEA TF target identification feature [C3‐TFT (all transcription factor targets, 1,127 gene sets)] on the differentially abundant mRNAs (Figure 1b) as a gene set input, in order to identify those TFs [normalized enrichment score (NES) >1.6 and *p*‐value <0.05] that might potentially coordinate their transcription. Interestingly, using these criteria, most of these putative regulatory TFs were members of either the forkhead box (FOX) or the homeobox (HOX) families (Appendix S3, *enriched TFs—linear model*). The proteins in both the FOX and HOX families are evolutionarily conserved and govern many processes during embryonic development and in adult life (Duverger & Morasso, 2008; Golson & Kaestner, 2016). Notably, we identified TFs previously associated with longevity, such as heat shock factor 1 (HSF1), forkhead box A [FOXA; also known as hepatocyte nuclear factor 3 (HNF3)], and paired box 4 (PAX4), as significantly enriched with age in our healthy cohort (Figure 2d), suggesting that they might putatively regulate transcription of several of the differentially expressed mRNAs. HSF1, through its influence on proteostasis, and the ortholog of FOXA in *Caenorhabditis elegans*, PHA‐4, were shown to modulate *C. elegans* life span (Hsu et al., 2003; Morley & Morimoto, 2004; Panowski et al., 2007), whereas PAX4 was associated with longevity in a Korean cohort (Park et al., 2009). Notably, interferon regulatory factor 1 (IRF1), a TF which governs innate immune responses by predominantly promoting the transcription of type‐I interferon genes and is implicated in cellular senescence (Feng et al., 2021; Frisch & MacFawn, 2020), was also identified as a putative transcriptional regulator of subsets of mRNAs enriched with age (Figure S2a).

In addition to the aforementioned TFs previously associated with longevity, we also found several FOX members [FOXJ1, FOXJ2, and FOXD3 (Figure 2e)], as well as HOX members [HOXA4, TGFB‐induced factor homeobox (TGIF), pre‐B‐cell leukemia homeobox 1 (PBX1), HNF6 (also known as OC‐1), HNF1A, MSX‐1, and pituitary‐specific positive transcription factor 1 (PIT1; also known as POU1F1) (Figure 2f and Figure S2b)] as predicted transcriptional regulators of several mRNAs changing with age. Whether these TFs coordinately control aging‐associated gene expression programs is unknown at present.

Nonetheless, while these TFs belong to two major families, FOX (Figure 2g) and HOX (Figure 2h), each factor has unique transcriptional targets based on the GSEA method (Appendix S4), supporting the view that a broad subset of differentially expressed transcripts is required for healthy aging. When employing the exclusive subset of differentially expressed transcripts (unadjusted *p*‐value <0.05) identified by the spline model, similar TFs were preferentially enriched based on the GSEA method (Appendix S3, *enriched TFs—spline model*). Notably, lymphoid enhancer‐binding factor 1 (LEF1), which shares homology with the high mobility group protein‐1 (HMGB1) (Giese et al., 1991), was among the most preferentially enriched TFs in our analysis (Figure S2c).

### 2.3 Linear lncRNAs following age‐dependent expression patterns are predicted targets of splicing RBPs

**FIGURE 3:**
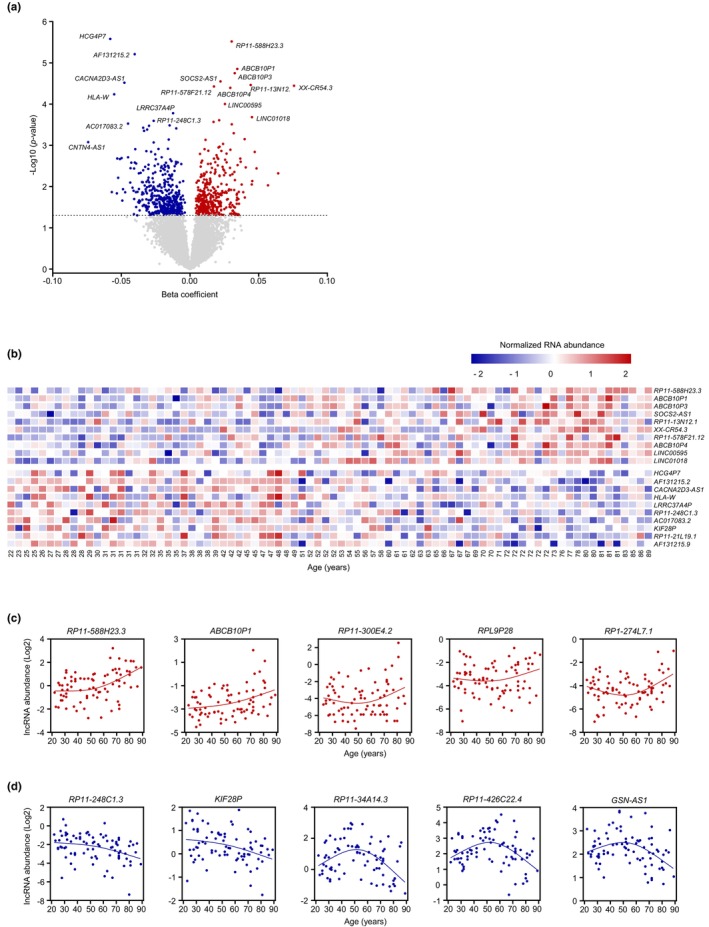
Differentially expressed linear lncRNAs as a function of age. (a) Volcano plot showing beta coefficients of linear lncRNAs expressed with age (per year). Transcripts showing significantly increased (red dots) or decreased (blue dots) levels with age (*p* < 0.05, adjusted for covariates) are indicated. Gray dots show transcripts that did not change significantly with age (*p* > 0.05). (b) Heatmap of the top 10 significantly elevated linear lncRNAs (*top*) and top 10 significantly reduced lncRNAs (*bottom*) with age based on a linear regression model (unadjusted *p*‐values). (c,d) Regression graphs of linear lncRNAs differentially increased (c) or decreased (d) with advanced aging based on a spline regression model (unadjusted *p*‐values). In (b), the *R* ^2^ values for the reported lncRNAs increasing with advancing age are as follows: *R* ^2^ = 0.17 for *RP11‐588H23.3*; *R* ^2^ = *0*.095 for *ABCB10P1*; *R* ^2^ = 0.11 for *ABCB10P3*; *R* ^2^ = 0.12 for *SOCS2‐AS1*; *R* ^2^ = 0.18 for *RP11‐13 N12.1*; *R* ^2^ = 0.17 for *XX‐CR54.3*; *R* ^2^ = 0.16 for *RP11‐578F21.12*; *R* ^2^ = 0.1 for *ABCB10P4*; *R* ^2^ = 0.15 for *LINC00595*; and *R* ^2^ = 0.17 for *LINC01018*. Similarly, for the reported transcripts decreasing with advancing age, the *R* ^2^ values are as follows: *R* ^2^ = 0.049 for *HCG4P7*; *R* ^2^ = 0.22 for *AF131215.2*; *R* ^2^ = 0.19 for *CACNA2D3‐AS1*; *R* ^2^ = 0.02 for *HLA‐W*; *R* ^2^ = 0.12 for *LRRC37A4P*; *R* ^2^ = 0.11 for *RP11‐248C1.3*; *R* ^2^ = 0.14 for *AC017083.2*; *R* ^2^ = 0.12 for *KIF28P*; *R* ^2^ = 0.085 for *RP11‐21 L19.1*; and *R* ^2^ = 0.21 for *AF131215.9*.

Given the rising interest in lncRNAs associated with aging and age‐related diseases (Grammatikakis et al., 2014; J. Kim et al., 2016), we first identified linear lncRNAs showing strong correlations with age in our study. Using a cutoff length of >200 nucleotides, we identified a total of 11,570 lncRNAs in this cohort; among them, 800 were differentially abundant with advancing age using the linear regression model and an unadjusted *p*‐value <0.05 (Figure 3a and Appendix S5, *linear lncRNAs—linear model*). The top differentially expressed linear lncRNAs (linear model) as a function of age are poorly characterized (Figure 3b). Using the same length cutoff criterion in the spline regression model, 728 lncRNAs were differentially abundant (unadjusted *p*‐value <0.05) with age (Appendix S5, *linear lncRNAs—spline model*). Here too, most lncRNAs were differentially abundant with age whether we used linear or spline regression analysis; for a few of them, the slope increased (Figure 3c) or decreased (Figure 3d) after 50 years of age and thus were detected only using spline regression analysis.

As studied for mRNAs (Figure S1), we sought to identify if the abundance of linear lncRNAs changed in female and male participants with advancing age. By linear regression analysis (unadjusted *p*‐value <0.05), we identified 730 and 475 differentially expressed linear lncRNA with age in females (Figure S3a and Appendix S5, *lncRNAs—linear—females*), and males (Figure S3b and Appendix S5, *lncRNAs—linear—males*), respectively. As observed for mRNAs, with the spline model the number of differentially expressed linear lncRNAs (unadjusted *p*‐value <0.05) increased markedly to 1723 in females (Figure S3c and Appendix S5, *lncRNAs—spline—females*), whereas only 279 linear lncRNAs were differentially expressed in males (Figure S3d and Appendix S5, *lncRNAs—spline—males*).

**FIGURE 4:**
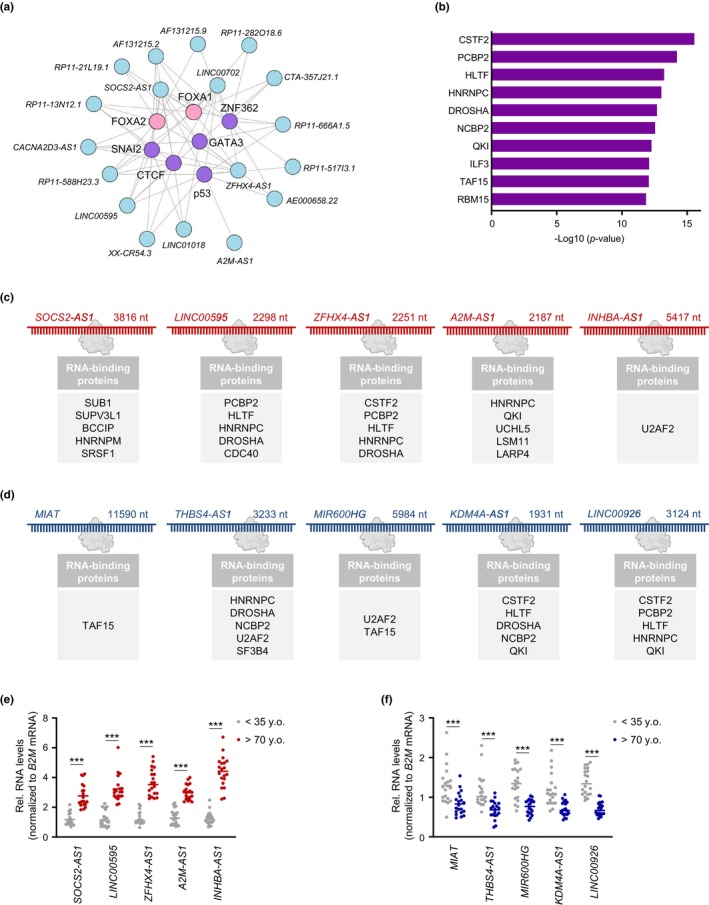
TFs capable of transcribing and RBPs capable of binding to linear lncRNAs differentially abundant with age based on a linear regression model. (a) TFs FOX (pink) or zinc‐finger (purple) putatively interacting with top differentially expressed linear lncRNAs (*p* < 0.05) based on the LncSEA method. (b) RBPs putatively interacting with linear lncRNAs based on LncSEA. (c,d) List of RBPs predicted to interact with differentially increased (c) or decreased (d) lncRNAs in our LncSEA. (e,f) Validation of differentially increased (e) or decreased (f) linear lncRNAs using RT‐qPCR analysis. Data were normalized to *B2M* mRNA, encoding a housekeeping protein. Significance was established using Student's *t* test. *** *p* < 0.001.

To better understand why these linear lncRNAs were differentially expressed as a function of age (adjusted for all covariates, unadjusted *p*‐value <0.05), we sought to identify preferentially enriched TFs interacting predominantly with the promoter region of these linear lncRNAs using the LncSEA method (transcription factor feature). Utilizing the differentially abundant linear lncRNAs detected using the linear regression model (Appendix S5, *linear lncRNAs—linear model*), we identified a number of zinc‐binding TFs, including snail family transcriptional repressor 2 (SNAI2), GATA‐binding protein 3 (GATA3), zinc finger protein 362 (ZNF362), CCCTC‐binding factor (CTCF), and p53 (Cassandri et al., 2017; Nicolai et al., 2015), predicted to bind to the promoter regions of these lncRNAs (*p* < 0.05; Appendix S6, *enriched TFs—linear model*). Surprisingly, members of the FOX family (FOXA1 and FOXA2) were once again preferentially enriched in our analysis, thus highlighting the potential importance of FOX TFs in healthy aging (*p* < 0.05; Appendix S6, *enriched TFs—linear model*). Notably, these TFs shared lncRNA targets, thus potentially implying that these linear lncRNAs are transcriptionally regulated by multiple TFs as a function of age (Figure 4a). Strikingly, although most TFs were shared regardless of the regression model used to detect differentially abundant linear lncRNAs, the TF MYC was more deeply enriched when using the subset of linear lncRNAs detected by the spline regression model (Appendix S6, *enriched TFs—spline model*).

Given that there is virtually no information on the roles of most linear lncRNAs differentially expressed in our healthy cohort and that RBP‐lncRNA interactions are closely linked to lncRNA function (Herman et al., 2022), we sought to identify enriched interacting RBPs by using the RBP feature in the LncSEA platform. Focusing on the linear lncRNAs differentially abundant with age by linear regression analysis (unadjusted *p*‐value <0.05), we found several proteins implicated in RNA splicing, including poly(C)‐binding protein (PCBP2), heterogeneous nuclear ribonucleoprotein C (HNRNPC), and RNA‐binding motif protein 15 (RBM15) (Georgiadou et al., 2021; Zarnack et al., 2013; L. Zhang et al., 2015), as being preferentially enriched by the LncSEA method (*p* < 0.05) with age (Figure 4b and Appendix S7, *enriched RBPs—linear model*). Notably, after applying the differentially expressed transcripts detected by the spline regression model to the same analysis, we identified other RBPs that also affected RNA splicing, including FAS‐activated serine/threonine kinase D2 (FASTKD2), which governs mitochondrial RNA processing and translation (Popow et al., 2015) (Figure S3e and Appendix S7, *enriched RBPs—spline model*).

The top elevated and reduced lncRNAs (from Appendix S5) known to interact with the RBPs in Figure 4b and Appendix S7 based on crosslinking analysis available through the LncSEA platform are indicated (Figure 4c,d). RT‐qPCR analysis was used to validate the levels of five annotated lncRNAs that increased (Figure 4e; *SOCS2‐AS1*, *LINC00595*, *ZFHX4‐AS1*, *A2M‐AS1*, and *INHBA‐AS1*) or decreased (Figure 4f; *MIAT*, *THBS4‐AS1*, *MIR600HG*, *KDM4A‐AS1*, and *LINC00926*) in old (>70 years old, y.o.) relative to young individuals (<35 y.o.).

### 2.4 Differentially expressed circRNAs as a function of donor age in human skin fibroblasts

**FIGURE 5:**
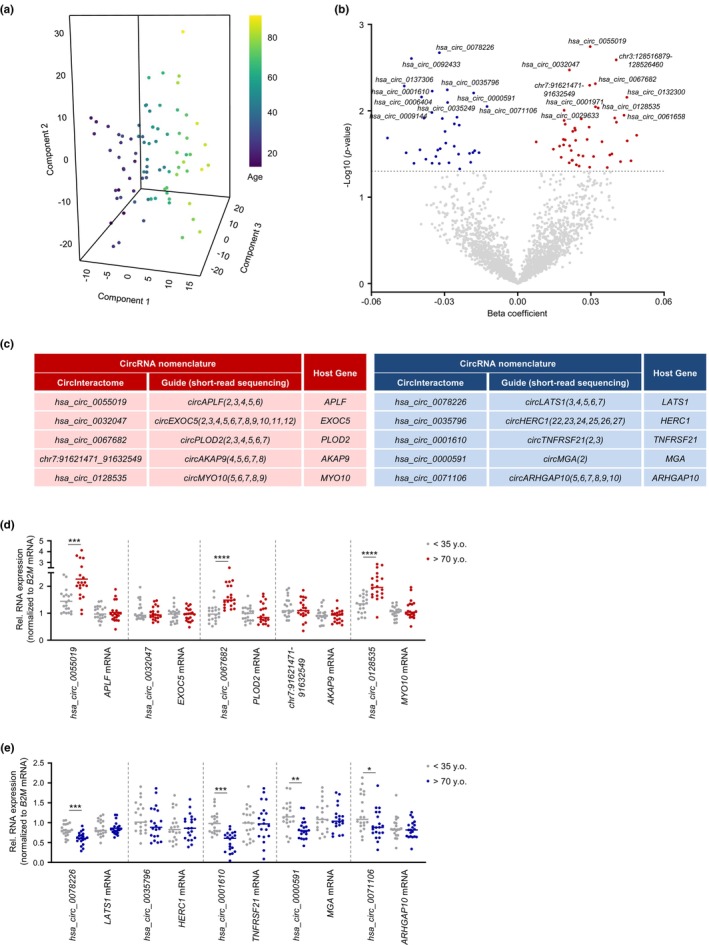
Differentially expressed circular lncRNAs as a function of age. (a) PLS analysis of age‐associated circular lncRNAs. (b) Volcano plot showing beta coefficients of circular lncRNAs expressed with age (per year). Transcripts showing significantly enhanced (red dots) or reduced (blue dots) levels with age (*p* < 0.05, adjusted for covariates) are indicated. Gray dots show transcripts that did not change significantly with age (*p* > 0.05). (c) Nomenclatures of increased (*left table*) or decreased (*right table*) circular lncRNAs and the respective cognate linear mRNAs. (d,e) Validation by RT‐qPCR analysis of differentially increased (d) or decreased (e) circular lncRNAs. Data were normalized to *B2M* mRNA, encoding a housekeeping protein. Significance was established using Student's *t* test. * *p* < 0.05, ** *p* < 0.01, *** *p* < 0.001.

To complete the characterization of differentially expressed RNAs, we assessed the differential expression of circular lncRNAs (circRNAs) with age. Excluding small circRNAs (<200 nts) from further analysis, we detected 46,120 circRNAs with at least one junction count in any donor. After including the additional requirement that at least seven donors within any age group should have at least one junction count for each specific circRNA, we focused on 2345 circRNAs (Appendix S8). In this circRNA pool, supervised partial least square (PLS) analysis revealed distinct separation across the age groups based on circRNA signatures in our cohort (Figure 5a). Linear regression analysis of this set revealed 47 elevated and 37 decreased circRNAs (unadjusted *p‐*value <0.05) across aging (Figure 5b); notably, two of the increased circRNAs, *chr3:128516879_128526460* and *chr7:91621471_91632549*, were considered novel as their junctions have not been reported in the major databases. After considering the relative abundance (baseMean >1.5), significance (*p* < 0.01), and beta coefficient with age (|beta coefficient| >0.01), we then focused on validating the top five circRNAs in each group, as well as the cognate linear mRNAs sharing the same precursor transcripts, in young (<35 y.o.) and old (>70 y.o.) donors using RT‐qPCR analysis; the nomenclatures of these circRNAs (Figure 5c) are based on CircInteractome (Dudekula et al., 2016) and a new guide to naming eukaryotic circRNAs (L. L. Chen et al., 2023). We used the nomenclature guide and predicted circRNA body composition based on short‐read sequencing.

Among those circRNAs elevated in older individuals, we successfully validated three transcripts with age‐dependent increased expression, *hsa_circ_0055019*, *hsa_circ_0067682*, and *hsa_circ_0128535* (Figure 5d), whereas *hsa_circ_0078226*, *hsa_circ_0001610*, *hsa_circ_0000591*, and *hsa_circ_0071106* were significantly lower in older donors (Figure 5e); interestingly, none of the tested linear cognate mRNAs changed significantly with age. Given the modest changes observed in the validated circRNAs, analyzing pools of circRNAs, rather than individual circRNAs, may be more informative in this paradigm. When assessing the sex differences in the expression of circRNAs, we only found 29 increased and 43 decreased circRNAs in females (unadjusted *p*‐value <0.05; Figure S4a and Appendix S8, *circular lncRNAs—females*), and only 41 increased and 14 decreased in males (unadjusted *p*‐value <0.05; Figure S4b and Appendix S8, *circular lncRNAs—males*) as a function of age.

## 3 DISCUSSION

There has been a recent surge of interest in identifying the molecular gene expression programs (RNAs and proteins) that govern aging physiology and disease. The analysis of human tissues and primary cells from healthy individuals can be particularly informative about the mechanisms that modulate healthy aging, with the ultimate goal of improving mean and maximum life span and health span. We recently employed an ex vivo model of primary skin fibroblasts to identify altered pathways across the life span in a healthy human cohort (GESTALT, NIA, NIH) (Tsitsipatis et al., 2022). Here, we sought to expand our understanding of these cells by analyzing their transcriptomes.

Using high‐throughput RNA‐seq analysis, we identified the lncRNAs (linear and circular) and mRNAs that were differentially abundant as a function of age in this healthy cohort employing both linear and spline regression models. Although most differentially abundant mRNAs and lncRNAs were detected using both models (Appendixes [Link], [Link], [Link]), a subset of these RNAs was only found using the spline model. This trend was even more evident when we analyzed separately female and male participants (Figures S1 and S3a–d). As shown, in females substantially more mRNAs and linear lncRNAs were identified by the spline model than the linear model (Figures S1c and S3c), whereas in males the number of mRNAs and linear lncRNAs was comparable, whether we used the linear or spline model (Figures S1d and S3d). This observation is in line with reports suggesting possible nonlinear changes in skin thickness (Shuster et al., 1975), the secretome (Lehallier et al., 2019), and DNA methylation (Vershinina et al., 2021) with age, further suggesting that integrating both linear and spline models is important when studying age‐related changes. Notably, several reports suggest that changes in skin are associated with hormonal imbalances fostered by menopause (Rahrovan et al., 2018; Windhager et al., 2019), which in turn can lead to nonlinear changes (Brincat et al., 1983). We thus sought to identify how the transcriptome may change separately in females and males as a function of healthy aging.

A comparison between changes in the proteome (Tsitsipatis et al., 2022) and the coding transcriptome (mRNAs) of the same cohort with age revealed modest correlations between altered mRNA and protein levels as a function of age (Figure 2a). It is worth noting that many proteins were differentially abundant with age without corresponding changes in the levels of the encoding mRNAs (Figure 2c) and, conversely, many mRNAs showed altered abundance without corresponding changes in protein levels (Figure 2b). Such discrepancies can be attributed to highly regulated processes like alternative splicing to produce mRNA variants, as well as to changes in translation efficiency, protein processing, protein stability, protein secretion, etc., which warrant consideration when assessing changes in the levels of proteins and corresponding mRNAs (Koussounadis et al., 2015; Mertins et al., 2016). These discrepancies also underscore the caution that must be exercised when assuming that changes in mRNA levels are a surrogate for changes in the levels of the encoded proteins or the functional pathways governed by such proteins.

Strikingly, many of the most prominent mRNAs were predicted to be transcriptionally regulated by TF members of the FOX and HOX families (Figure 2d–h and Appendix S2). Future studies are required to evaluate whether these TFs play prominent roles in skin aging, although there is information to suggest that they may be broadly involved in aging. For example, FOXJ1 has a key role in regulating the expression of genes important for ciliogenesis in primary human and mouse airway epithelial cells (Didon et al., 2013; You et al., 2004). Growing evidence suggests a prominent role for primary cilia as receiving or releasing organelles for extracellular vesicles (EVs) and as sensors of transduced signals (Hosio et al., 2020; Ikegami & Ijaz, 2021). With emerging interest in the role of EVs in aging and age‐related diseases (Takasugi, 2018; Yin et al., 2021), whether FOXJ1 elicits a similar role in regulating ciliogenesis in skin fibroblasts and in turn affects the uptake or release of EVs with age, warrants further investigation. Another interesting TF that was among the most preferentially enriched TFs in our analysis, LEF1, shares homology with HMGB1, a protein that translocates from the nucleus to the extracellular space, has a role in cellular senescence, and fine‐tunes the skin macroenvironment to enhance wound healing (Davalos et al., 2013; Phan et al., 2020; Sofiadis et al., 2021). Whether LEF1 may have a role in senescence or wound healing remains to be studied. Notably, the levels of these TFs and RBPs did not significantly change with age in our previous proteomic analysis, likely indicating that mechanisms affecting their activity (not necessarily their levels), such as posttranslational modifications or shuttling across the nuclear envelope, may be more prominent in these cells.

We identified a number of differentially expressed linear lncRNAs (Figure 3) that were not previously identified as being associated with advanced aging; among the validated lncRNAs, *SOCS2‐AS1* was proposed to sponge microRNAs in tumor progression models (Jian et al., 2021; Zheng et al., 2020), and *MIAT* controls the development of the atherosclerotic lesion and plaque destabilization in atherosclerosis (Fasolo et al., 2021). Strikingly, the levels of *SOCS2* and *INHBA* mRNAs, transcribed from loci near those of the respective lncRNAs, were significantly elevated with age, whereas the levels of *THBS4* mRNA were reduced, potentially suggesting that *SOCS2‐AS*, *INHBA‐AS*, and *THBS4‐AS* may act as *cis*‐regulatory elements. FOXA1 and FOXA2, two members of the FOX family, were predicted by LncSEA to interact with the promoters of the differentially expressed linear lncRNAs (Figure 4a), supporting the notion that FOX TFs may foster programs underlying healthy aging by regulating the expression of both coding and long noncoding transcripts. Besides FOX TFs, TF members of the zinc finger family were identified as preferentially interacting with the promoters of a wide subset of differentially expressed linear lncRNAs showing age‐related changes in expression in our analysis. Given the emergence of zinc finger TFs as prominent antiaging targets (Fischer et al., 2022; Zimmermann et al., 2019), activating the transcription of lncRNAs may be part of the healthy aging program elicited by zinc finger TFs. Notably, many of the differentially expressed linear lncRNAs were preferentially associated with RBPs implicated in RNA splicing (Figure 4b and Figure S3e). With escalating interest in the potential role of splicing in healthy aging and longevity (Angarola & Anczukow, 2021; Bhadra et al., 2020), whether the interactions of the linear lncRNAs or mRNAs with the RBPs predicted in our analysis have a prominent role in healthy aging warrants further investigation.

While the roles of the vast majority of circRNAs are poorly understood, a few of the validated circRNAs in our study were previously associated with age‐related declines or disease. For example, *hsa_circ_0000591* and *hsa_circ_0071106* were previously linked to knee osteoarthritis and type 2 diabetes, respectively (Jiang et al., 2021; Yingying et al., 2021), whereas delivery of *hsa_circ_0001610* via EVs from tumor‐associated macrophages reduced radiosensitivity in endometrial cancer (Gu et al., 2021). Notably, we identified the RBP interleukin enhancer‐binding factor 3 (ILF3) as interacting with linear lncRNAs that changed with age (Figure 4b), and ILF3 coordinates the biogenesis of some circRNAs during viral infection (Li et al., 2017). In light of emerging interest in the expression of circRNAs in healthy aged individuals, supervised PLS analysis of differentially abundant circRNAs showed a prominent age‐dependent distribution in this cohort (Figure 5a). The fact that linear RNAs revealed a less apparent distribution with age (not shown) suggests that circRNAs may track better with human age, particularly when jointly analyzing groups of circRNAs. Similar studies are needed to test if circRNA analysis can be informative in instances of age‐associated diseases.

To conclude, it is worth noting that the study design may cause spurious correlations and that the stringent criteria to recruit healthy individuals inevitably reduced the size of our cohort. Although some of the observed changes were validated using molecular biology techniques (Figure 4e,f and Figure 5d,e), future studies that include larger numbers of participants are needed to validate and extend the current findings. Also, given that the cultured primary skin fibroblasts do not faithfully recapitulate all traits of the aging human skin, where cells are typically quiescent and exposed to different endogenous factors, it will be important to complement our studies with single‐cell RNA‐seq analysis (Sole‐Boldo et al., 2020; Zou et al., 2021) and spatial transcriptomic analysis of aging skin. While these approaches enable the study of skin cells closer to their native state without artifacts arising from cell culture, they mainly detect the most abundant RNAs. With growing appreciation of the key roles of low‐abundance linear and circular lncRNAs in driving protein programs and cell fate (Herman et al., 2022), improving and integrating methods of analysis of all RNA molecules changing with advancing age will lead to a better understanding of the molecular mechanisms that drive healthy aging.

## 4 METHODS

### 4.1 Skin biopsies and culture of skin fibroblasts

Skin biopsies and expanded fibroblast cultures were obtained as previously described (Tsitsipatis et al., 2022). Briefly, punch skin biopsies (4 mm^2^) were obtained from the non‐sun‐exposed skin of the inner upper arm, just below the axilla, of 82 healthy GESTALT participants (Table 1) following a stringent clinical protocol that minimized the risk of infections and side effects (Tanaka et al., 2018). The enrolled participants were free of any major diseases, did not take any prescribed medication except a single monotherapy for hypertension, had no physical or cognitive impairments, did not train professionally, and had a body mass index (BMI) less than 30 kg/m^2^. The inclusion criteria were assessed during a 6‐h evaluation at the Clinical Research Unit (NIA IRP) based on medical history, physical exams, and blood tests interpreted by an experienced nurse practitioner (Roy et al., 2022; Schrack et al., 2014). Collected skin biopsies were minced into smaller pieces and distributed into three wells of 6‐well, collagen‐coated plates. The minced biopsies were incubated in Dulbecco's Modified Eagle Medium (DMEM, Gibco) supplemented with 20% fetal bovine serum (Gibco), 1% penicillin–streptomycin (Gibco), and 1% nonessential amino acids (Gibco) at 37°C in a humidified atmosphere for 2 weeks; then in DMEM supplemented with 10% FBS, 1% penicillin–streptomycin, and 1% nonessential amino acids for 1–2 weeks until confluent. The established primary human dermal fibroblasts (HDFs) were further expanded to three 100‐mm culture plates, grown until confluency, and frozen until use. The GESTALT protocol is approved by the Intramural Research Program of the US National Institute on Aging and the Institutional Review Board of the National Institutes of Health. All participants provided written, informed consent at every visit.

### 4.2 RNA isolation, library preparation, and RNA sequencing

After thawing, HDFs were cultured in DMEM supplemented with 10% FBS, 1% penicillin–streptomycin, and 1% nonessential amino acids at 37°C in a humidified atmosphere until they reached confluency and were used within four passages. Cells were harvested, washed once with 1× PBS, and total RNA was isolated using the Direct‐zol™ RNA MiniPrep kit (Zymo Research) following the manufacturer's instructions. Following RNA isolation, quality was assessed on an Agilent Bioanalyzer, and 500 ng of total RNA was subjected to Ribo‐RNA depletion with Low Input RiboMinus Eukaryote System v2 (Thermo Fisher Scientific). Ribo‐RNA‐depleted samples were then used for cDNA synthesis with Ovation® RNA‐Seq System V2 (Nugen) following the manufacturer's protocol. Briefly, the first cDNA strand was synthesized from Ribo‐RNA‐depleted samples using a unique first strand DNA/RNA chimeric primer mix and reverse transcriptase (RT) included in the kit, followed by synthesis of the second cDNA strand. After purification with Agencourt RNA CleanUp XP beads, the double‐stranded cDNA products were amplified with the Single primer isothermal amplification (SPIA) included in the kit. The amplified products were then purified with QIAGEN QIAquick PCR Purification Kit (QIAGEN) and checked on an Agilent 2100 Bioanalyzer with a DNA 1000 kit (Agilent) and fragmented by a Bioruptor. Fragmented cDNAs were checked again on Agilent 2100 Bioanalyzer with a DNA 1000 kit.

The fragmented cDNAs were used for library preparation with Illumina TruSeq ChIP Library Preparation Kit (Illumina) according to the manufacturer's protocol. Briefly, the cDNAs were subjected to end repair, the 3′ end adenylation, and adapter ligation, and were then purified with AMPure beads (Beckman). The products were size‐selected with SPRIselect beads (Beckman), and then the selected cDNAs were amplified by PCR and purified again with SPRIselect beads to generate final libraries. Paired‐end sequencing was performed by Quick Biology (Pasadena), aiming for 250 million reads per sample using an Illumina NovaSeq 6000 instrument.

### 4.3 Reverse transcription (RT) followed by real‐time quantitative (q)PCR analysis

Following RNA isolation, 1 μg of total RNA was used for reverse transcription (RT) followed by real‐time quantitative PCR (qPCR) analysis. For qPCR analysis, 0.1 μL cDNA was used with 250 nM of primers (Appendix S9) and KAPA SYBR® FAST qPCR Kits (KAPA Biosystems) as described (Tsitsipatis et al., 2021). Divergent primers spanning the circRNA junctions of interest were designed using CircInteractome (Dudekula et al., 2016). RT‐qPCR analysis was carried out on a QuantStudio 5 Real‐Time PCR System (Thermo Fisher Scientific) with a cycle setup of 3 min at 95°C, 40 cycles of 5 s at 95°C, and 20 s at 60°C. Relative RNA levels were calculated after normalizing to beta‐2‐microglobulin (*B2M*) mRNA using the 2^−ΔΔCt^ method; among the examined mRNAs encoding housekeeping proteins, *B2M* mRNA levels showed the least variability across participants (not shown).

### 4.4 Bioinformatic and statistical analyses

Binary Base Call (BCL) files were demultiplexed and converted to FASTQ files using bcl2fastq program (v2.20.0.422). FASTQ files were trimmed for adapter sequences using Cutadapt version v1.18 and aligned to human genome hg19 Ensembl v82 using STAR software v2.4.0j (Dobin et al., 2013); featureCounts (v1.6.4) (Liao et al., 2014) were used to create gene counts from the samples for linear RNA analysis. The chimeric junction file obtained from STAR software was parsed for fusion junctions and analyzed using CIRCexplorer v1.1.10 (X. O. Zhang et al., 2014) to obtain the circularizing junction counts for circRNA analysis as well as for circRNA annotation. To filter out RNAs with very low counts across the cohort we divided the 82 samples into four groups, each enclosing approximately 20 age‐consecutive samples. We required at least seven samples in any group to have 10 or more counts for linear RNA analysis, whereas for the circRNA analysis one or more counts for a specific circRNA was required. Age‐related differential expression analysis for both linear and circular RNAs was conducted using the R Bioconductor package, DESeq2, version 1.36.0 (Love et al., 2014) after adjusting for gender and collection batches. The linear regression model was run using default parameters. Briefly, we applied default DESeq(), which uses the median ratio method, to estimate the size factors. Next, the dispersion was estimated assuming a negative binomial distribution for count, the model was fitted, and the Wald test was employed for significance testing (Anders & Huber, 2010). Significance was determined using *p*‐value <0.05 for all transcripts. For the spline model, we used natural spline with degree of freedom equal to three, and the spline model was run using the likelihood ratio test. The full model included the three spline covariates, along with batch and sex, while the reduced model included only batch and sex. The data on differentially abundant mRNAs and (linear or circular) lncRNAs as a function of age are summarized in Appendixes [Link], [Link], [Link].

For downstream plot generation, including heatmap and regression plots, we normalized and log‐transformed the count matrix using edgeR's (version 3.38.4) (Robinson et al., 2010) calcNormFactors and cpm functions. Next, adjusted count data were generated via the function “removeBatchEffect” in the LIMMA package (version 3.52.4) (Ritchie et al., 2015). For PLS plotting, we used adjusted data and the packages pls (version 2.8–1) as well as plotly (4.10.1). Count data for downstream plot generation, including PLS plot and heatmap, was created via the function “removeBatchEffect” in the LIMMA package (version 3.52.4) and Package plotly (4.10.1) was used for PLS plotting. GSEA of “transcription factor targets” gene set from Molecular Signature Database (https://www.gsea‐msigdb.org) was performed with GSEA_4.2.3 software (Subramanian et al., 2005) on C3‐TFT (all transcription factor targets, 1127 gene sets) feature using differentially expressed mRNAs (*p* < 0.05), whereas LncSEA (http://bio.liclab.net/LncSEA/) was run with either the “Transcription Factor” or the “RNA Binding Protein” feature using differentially expressed linear lncRNAs (*p* < 0.05) (J. Chen et al., 2021). The data generated by GSEA are summarized in Appendixes [Link], [Link], and the data from LncSEA are summarized in Appendixes [Link], [Link].

For RT‐qPCR analysis, quantitative data were represented as the means ± SD of the number of samples indicated in each case; statistical significance was established using unpaired Welch's *t* test in GraphPad Prism (9.0). A *p*‐value of <0.05 was considered statistically significant; significance was indicated in the figures as * *p* < 0.05, ** *p* < 0.01, *** *p* < 0.001. Graphs were generated using GraphPad Prism (9.0).

## AUTHOR CONTRIBUTIONS

DT, LF, and MG conceived the study; DT, JLM, and MG designed experiments; DT, JLM, KMM, ABH, YP, JHY, CA, MWC, LC, RM, and NB performed and analyzed experiments; XY, KA, CUM, and SD contributed intellectually and provided technical support; CWC, ACK, LZ, JD, MK, and LF collected the human biopsies; DT, JLM, KMM, LF, and MG wrote the manuscript.

## FUNDING INFORMATION

This research was supported in full by the National Institute on Aging Intramural Research Program of the National Institutes of Health.

## CONFLICT OF INTEREST STATEMENT

The authors declare that they have no conflicts of interest.

## Supporting information


Figures S1–S4

Appendix S1

Appendix S2

Appendix S3

Appendix S4

Appendix S5

Appendix S6

Appendix S7

Appendix S8

Appendix S9

## Data Availability

The RNA‐seq data were deposited in GEO (GSE226189).
