# Supplementary material for: Transcriptomes of human primary skin fibroblasts of healthy individuals reveal age‐associated mRNAs and long noncoding RNAs
Source: Aging Cell. 2023 Jul 18;22(11):e13915. doi: 10.1111/acel.13915 (PMC10652340; doi:10.1111/acel.13915)
Supplement: Supplementary file 1 — Figures S1–S4 [file ACEL-22-e13915-s010.pdf]

LEGENDS FOR SUPPLEMENTAL FIGURES AND DATASETS

FIGURE S1. Differentially expressed mRNAs in females and males as a function of age. (a, b) Volcano plot showing beta coefficients of mRNAs expressed with age (per year) in females (a) or males (b). mRNAs showing significantly increased (red dots) or decreased (blue dots) levels with age ($p < 0.05$). Gray dots show mRNAs that did not change significantly with age ($p > 0.05$). **(c, d)** Bar graphs showing the number of differentially expressed mRNAs in females (c) or males (d) using the linear regression or spline model.

FIGURE S2. Additional transcription factors putatively driving the expression of subsets of differentially abundant mRNAs with advanced aging. (a, b) GSEA of IRF1 (a) or transcription factors in the HOX family (b), enriched with advanced aging based on the differentially expressed mRNAs detected by linear regression model. **(c)** GSEA of LEF1 enriched with advanced aging based on the differentially expressed mRNAs detected by spline regression model.

FIGURE S3. Differentially expressed linear lncRNAs in females and males and enriched RNA-binding proteins interacting with differentially expressed linear lncRNAs with age based on spline regression model. (a, b) Volcano plot showing beta coefficients of linear lncRNAs expressed with age (per year) in female (a) and male (b) participants. LncRNAs showing significantly increased (red dots) or decreased (blue dots) levels with age ($p < 0.05$). Gray dots show lncRNAs that did not change significantly with age ($p > 0.05$). **(c, d)** Bar graphs showing the number of differentially expressed linear lncRNAs in females (c) or males (d) using the linear regression or spline model. **(e)** RNA-binding proteins interacting with linear lncRNAs, identified as being enriched by LncSEA analysis.

FIGURE S4. Differentially expressed circular lncRNAs in females and males with age. (a, b) Volcano plot showing beta coefficients of circular lncRNAs expressed with age (per year) in females (a) or males (b). Transcripts showing significantly increased (red dots) or decreased (blue dots) circular lncRNAs with age ($p < 0.05$). Gray dots indicate circular lncRNAs that did not change significantly with age ($p > 0.05$).

SUPPLEMENTARY FILE S1. Coding RNAs. Differentially expressed mRNAs using the linear and spline regression models. To take into consideration age-related trajectories, data were also analyzed separately for female and male participants.

SUPPLEMENTARY FILE S2. Overlap of mRNAs and proteins. Data used for establishing the correlations of the transcriptomic and proteomic analyses of the cohort.

SUPPLEMENTARY FILE S3. Enriched TFs differentially abundant mRNAs. Using the differentially expressed mRNAs, we performed GSEA to identify transcription factors which may regulate the expression of these prominent mRNAs.

SUPPLEMENTARY FILE S4. mRNAs putative targets of FOX and HOX. Differentially expressed mRNAs that were putative targets of forkhead box (FOX) and homeobox (HOX) transcription factors. Members of these two families were preferentially enriched based on GSEA reported in SUPPLEMENTARY FILE S3.

SUPPLEMENTARY FILE S5. Linear lncRNAs. Differentially expressed linear lncRNAs using the linear and spline regression models. To take into consideration age-related trajectories, data were also analyzed separately for female and male participants.

SUPPLEMENTARY FILE S6. Enriched TFs for differentially abundant linear lncRNAs. Using the differentially expressed linear lncRNAs, we used the LncSEA platform to identify transcription factors which may regulate the expression of these prominent linear lncRNAs.

SUPPLEMENTARY FILE S7. Enriched RBPs for linear lncRNAs. Using the differentially expressed linear lncRNAs, we used the LncSEA platform to identify RNA-binding proteins which may interact with these prominent linear lncRNAs.

SUPPLEMENTARY FILE S8. circular lncRNAs. Differentially expressed circular lncRNAs using the linear regression model. To take into consideration age-related trajectories, data were also analyzed separately for female and male participants.

SUPPLEMENTARY FILE S9. Primer pairs. Primer pairs employed in this study to validate the expression of lncRNAs (linear and circular) by using RT-qPCR analysis.

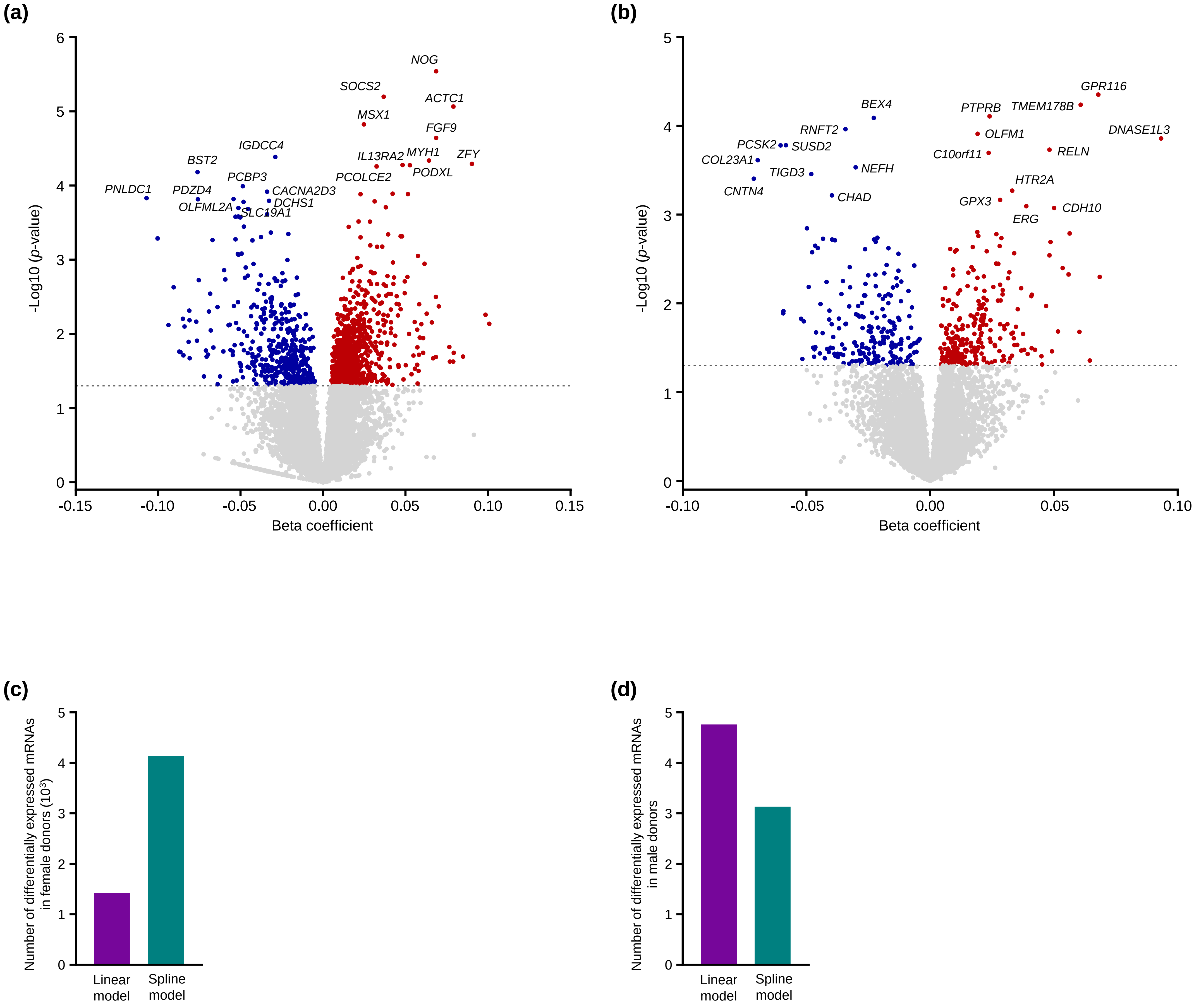
(a)
-Log10 (p-value)
Beta coefficient
NOG
SOCS2
ACTC1
MSX1
FGF9
IGDCC4
BST2
IL13RA2
MYH1
ZFY
PODXL
PCBP3
PCOLCE2
PNLDC1
PDZD4
CACNA2D3
DCHS1
OLFML2A
SLC19A1
(b)
-Log10 (p-value)
Beta coefficient
GPR116
BEX4
PTPRB
TMEM178B
RNFT2
OLFM1
DNASE1L3
PCSK2
SUSD2
C10orf11
RELN
COL23A1
TIGD3
NEFH
HTR2A
CNTN4
CHAD
GPX3
CDH10
ERG
(c)
Number of differentially expressed mRNAs in female donors (10³)
Linear model
Spline model
(d)
Number of differentially expressed mRNAs in male donors
Linear model
Spline model

(a)

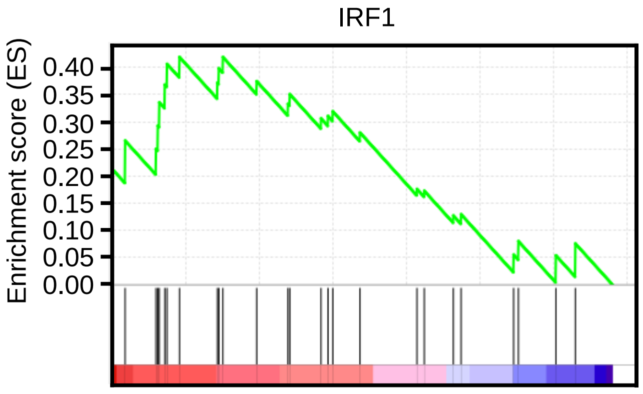

Nominal p-value	0.04009434
FDR q-value	0.39952198
FWER p-Value	0.999

(b)

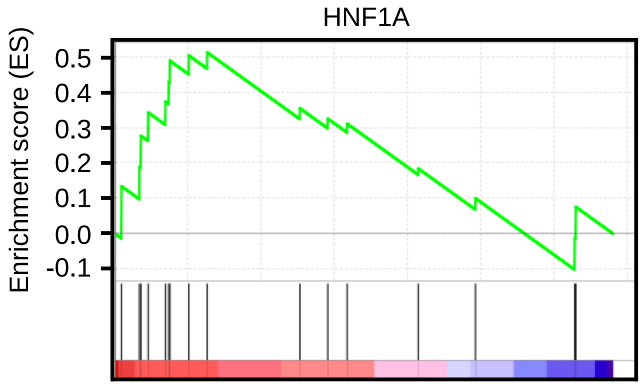

Nominal p-value	0.012658228
FDR q-value	0.41799435
FWER p-Value	0.982

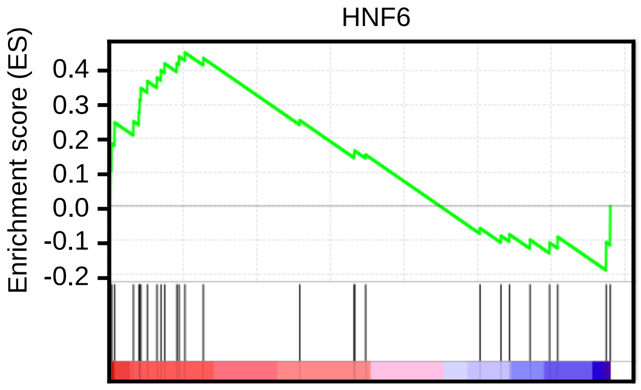

Nominal p-value	0.0125
FDR q-value	0.43913415
FWER p-Value	0.922

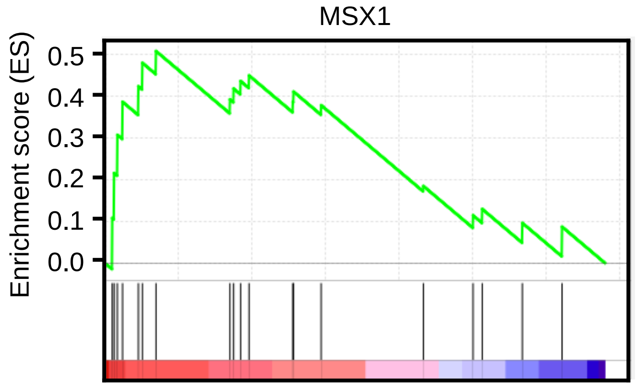

Nominal p-value	0.011389522
FDR q-value	0.47624522
FWER p-Value	0.914

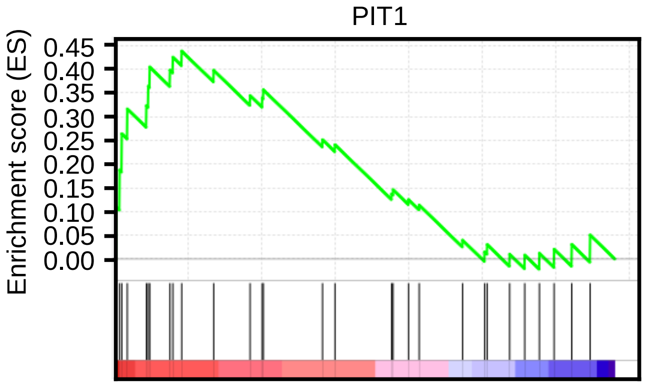

Nominal p-value	0.018648019
FDR q-value	0.40305927
FWER p-Value	0.931

(c)

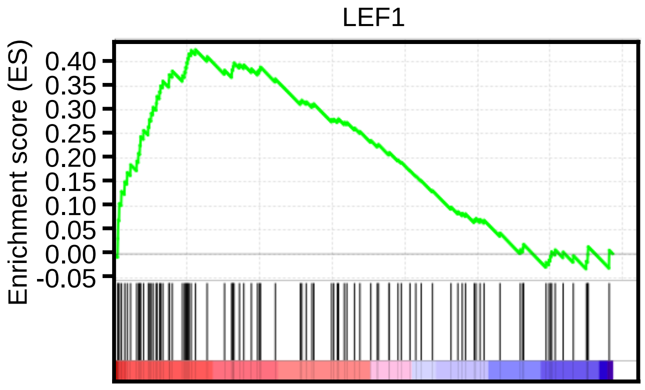

Nominal p-value	0.0
FDR q-value	0.08094915
FWER p-Value	0.446

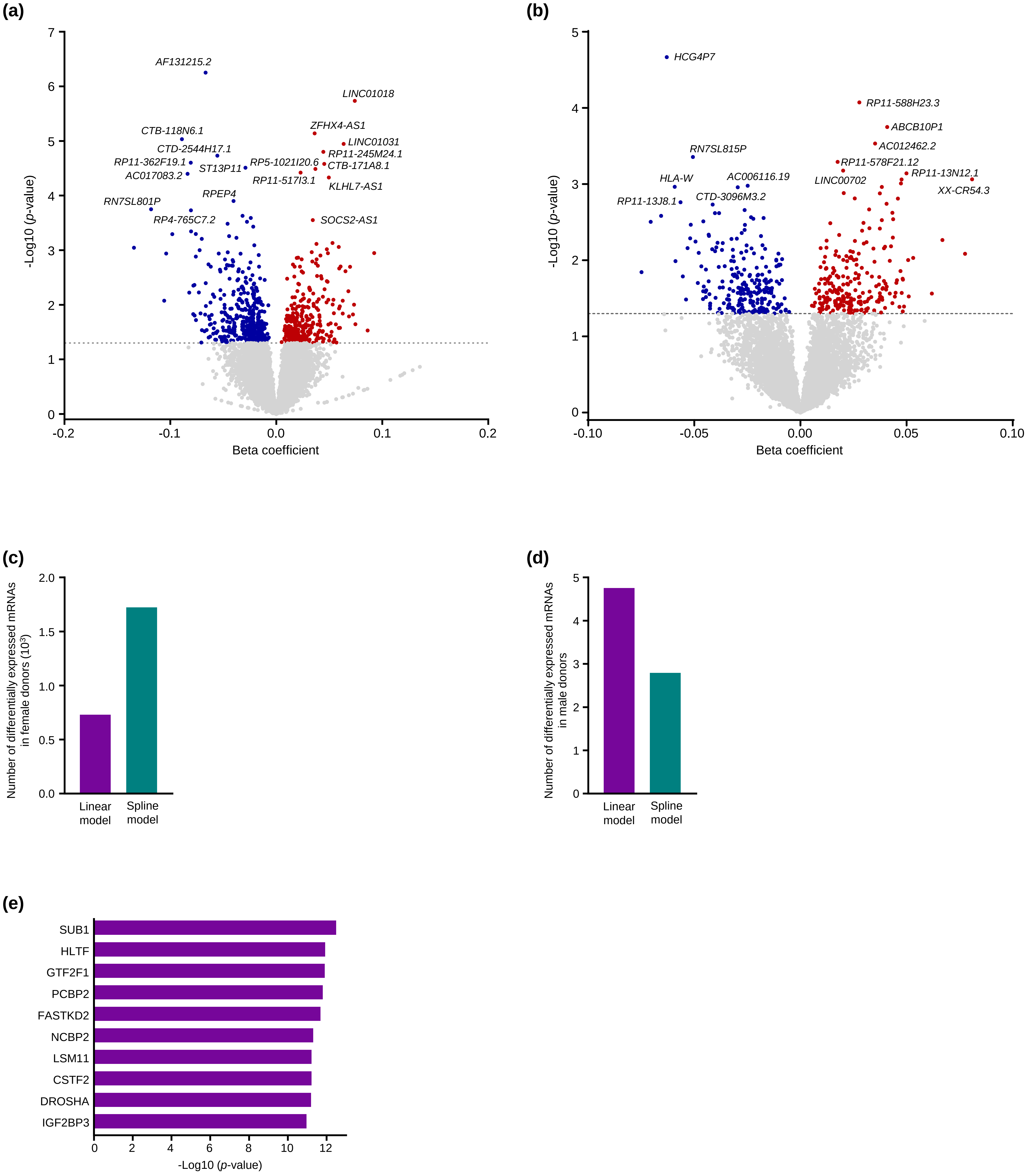
(a)
AF131215.2
LINC01018
CTB-118N6.1
ZFHX4-AS1
CTD-2544H17.1
LINC01031
RP11-245M24.1
RP11-362F19.1
RP5-1021I20.6
CTB-171A8.1
AC017083.2
ST13P11
RP11-517I3.1
KLHL7-AS1
RPEP4
RN7SL801P
RP4-765C7.2
SOCS2-AS1
-Log10 (p-value)
Beta coefficient
-0.2
-0.1
0.0
0.1
0.2
(b)
HCG4P7
RP11-588H23.3
ABCB10P1
RN7SL815P
AC012462.2
RP11-578F21.12
HLA-W
AC006116.19
RP11-13N12.1
LINC00702
XX-CR54.3
RP11-13J8.1
CTD-3096M3.2
-Log10 (p-value)
Beta coefficient
-0.10
-0.05
0.00
0.05
0.10
(c)
Number of differentially expressed mRNAs in female donors (10³)
Linear model
Spline model
(d)
Number of differentially expressed mRNAs in male donors
Linear model
Spline model
(e)
SUB1
HLTF
GTF2F1
PCBP2
FASTKD2
NCBP2
LSM11
CSTF2
DROSHA
IGF2BP3
-Log10 (p-value)

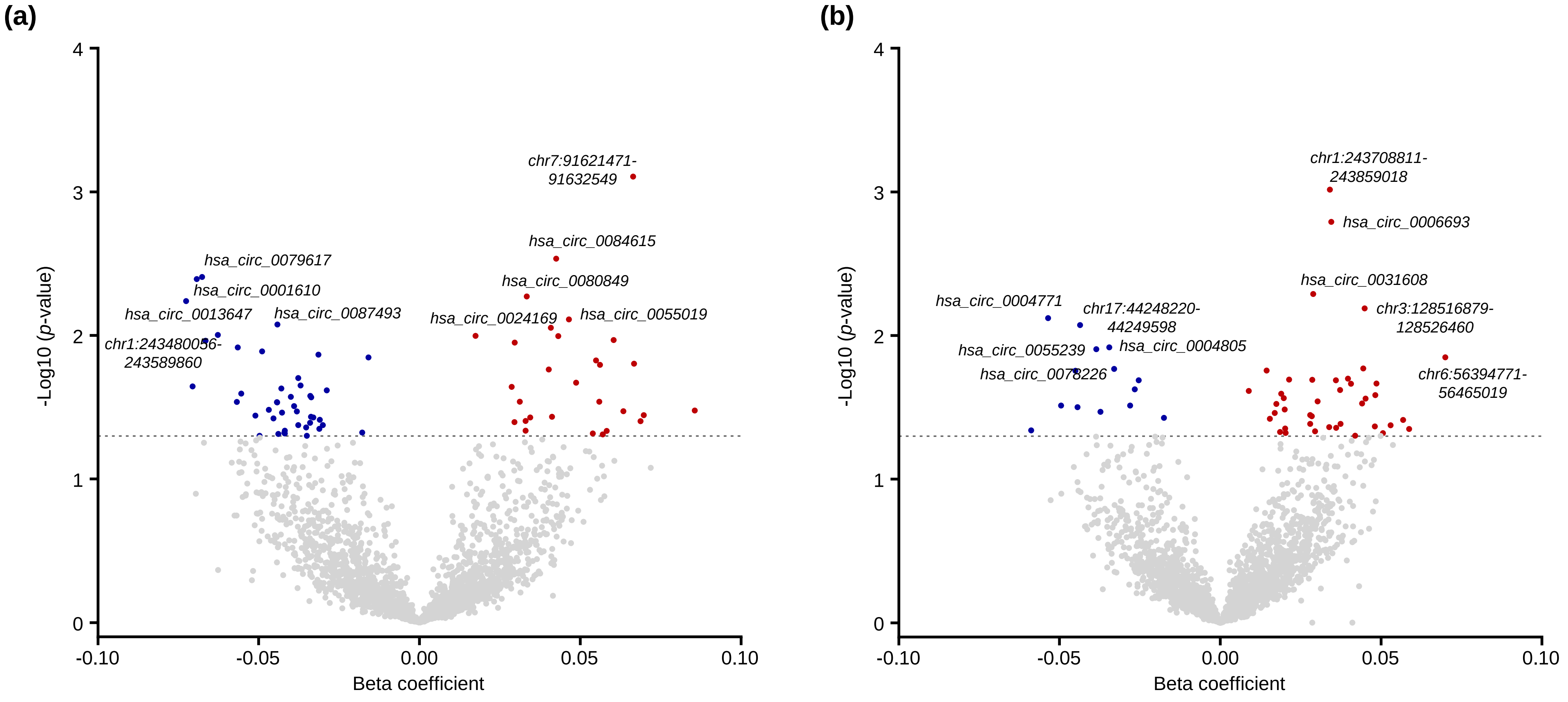
(a)
(b)
-Log10 (p-value)
Beta coefficient
0
1
2
3
4
-0.10
-0.05
0.00
0.05
0.10
chr7:91621471-91632549
hsa_circ_0084615
hsa_circ_0080849
hsa_circ_0024169
hsa_circ_0055019
hsa_circ_0079617
hsa_circ_0001610
hsa_circ_0013647
hsa_circ_0087493
chr1:243480056-243589860
chr1:243708811-243859018
hsa_circ_0006693
hsa_circ_0031608
chr3:128516879-128526460
chr6:56394771-56465019
hsa_circ_0004771
chr17:44248220-44249598
hsa_circ_0055239
hsa_circ_0004805
hsa_circ_0078226
